# Symptoms of Nocturnal Masticatory Muscle Activity among Women of Different Age Groups and Their Association to Obstructive Sleep Apnea—A Cross Sectional Study

**DOI:** 10.3390/jcm11051199

**Published:** 2022-02-23

**Authors:** Alona Emodi-Perlman, Jawan Soliman, Pessia Frideman-Rubin, Ilana Eli

**Affiliations:** Department of Oral Rehabilitation, The Maurice and Gabriela Goldschleger School of Dental Medicine, Sackler Faculty of Medicine, Tel Aviv University, Tel Aviv 6139001, Israel; jawan2903@gmail.com (J.S.); pessia80@gmail.com (P.F.-R.); elilana@tauex.tau.ac.il (I.E.)

**Keywords:** snoring, obstructive sleep apnea (OSA), sleep-disordered breathing (SDB), women, sleep bruxism (SB), masticatory muscle activity

## Abstract

Sleep bruxism (SB), snoring, and excessive daytime sleepiness are often associated with obstructive sleep apnea (OSA). OSA, which is characterized by a repetitive collapse of the upper airway during sleep, can cause oxygen desaturation and lead to adverse medical conditions, such as cardiovascular events, hypertension, heart attack, and stroke. In the present study, 112 Arab women aged 20–40 years (Early Adulthood/Adulthood–EarlyA) and 116 Arab women aged 50 and above (Middle Age–MiddleA), were requested to complete questionnaires regarding demographic variables, symptoms of nocturnal masticatory muscle activity (possible SB, headache, and stiffness of the oral and/or neck musculature upon awakening), risk of OSA (STOP-BANG questionnaire), and daytime sleepiness (Epworth sleepiness scale—ESS). Women, who reported snoring, experienced more SB (35.8% vs. 20.6%, chi-square, *p* < 0.05), more headaches (33.3% vs. 19.3%, *p* < 0.05), and more muscle stiffness upon awakening (34.3% vs. 16.3%, *p* < 0.005), than their non-snoring counterparts. Out of the women who snore, 11% showed high risk for OSA, as compared to only 1% among the non-snoring participants (*p* < 0.000). Symptoms of nocturnal masticatory muscle activity and/or snoring can serve as initial indicators of OSA. Higher awareness of dentists to such symptoms, particularly among their middle-aged female patients, can prevent a development of harmful conditions associated with OSA.

## 1. Introduction

Sleep-disordered breathing (SDB) is a vast term which includes conditions ranging from snoring to obstructive sleep apnea (OSA). On one side of the SDB spectrum, with high prevalence, lies the familiar symptom of snoring [1]. It increases with age [2] and it is known to be under-reported by women [3]. The other side of the SDB spectrum belongs to OSA, which is characterized by a repetitive collapse of the upper airway during sleep, often associated with oxygen desaturation and/or arousal from sleep [4]. OSA is more prevalent among men compared to women, even after correcting for age and body mass index [5]. For a state-of-the-art summary, regarding the epidemiology of OSA, refer to Young et al. [6].

In general, the risk of SDB among women increases after menopause [7,8,9]. Although nearly 20% of menopausal women experience sleep apnea [10], a vast majority of the women with sleep apnea do not acknowledge this problem [11,12]. Such under-reporting may lead to delay in diagnosis, an increase in the development of harmful conditions, such as cardiovascular events, hypertension, heart attack, stroke [13,14,15,16], and an increase in healthcare utilization [17].

Studies have shown that OSA is associated with several clinical signs and syndromes, such as snoring, excessive daytime sleepiness, and sleep bruxism (SB) [18,19,20]. Of the subjects with SB, 35% report snoring and about 17% present OSA. Among subjects with OSA, 30% complain of bruxism [21] which decreases after the treatment of OSA [22]. Such association between OSA and SB may happen due to frequent arousals following apneic episodes [23]. One hypothesis claims that SB plays a protective role by reopening compromised upper airways due to masticatory muscle contractions [24,25,26,27].

The aim of the study was to evaluate the risk of OSA among women belonging to different age groups and its relationship with signs of nocturnal masticatory muscle activity, snoring, and daytime sleepiness.

## 2. Materials and Methods

In a cross-sectional study, two distinct age groups of Israeli Arab women were evaluated. The rational for selecting these specific gender and age groups was to enable evaluation of the effect of menopause on the studied variables. 

Group 1: Arab women aged 20–40 years (Early Adulthood/Adulthood—EarlyA)

Group 2: Arab women aged 50 and above (Middle Age—MiddleA).

Subjects were recruited from four Arab villages in the North of Israel by two female Arab researchers (JS and a research assistant). Data were collected from mid-November until the end of December 2020.

Researchers approached subjects at their households. The aims and procedures of the study were explained to each participant personally, both in writing and orally. Special care was given when explaining the issue of sleep bruxism, mainly when clarifying the meaning of the terms “teeth clenching/grinding”.

Each participant was requested to complete self-report questionnaires, as detailed below. Subjects completed the questionnaires on the spot. A measurement of the subjects’ neck perimeter was carried out by one of the researchers, as detailed below.

The study was conducted in full accordance with the World Medical Association Declaration of Helsinki. The Ethics Committee of the Tel Aviv University approved all study procedures (0001711-1).

### 2.1. Instruments and Study Variables

#### 2.1.1. Demographic Information. Questions Referring to the Following Parameters

1. Age;
2. Height and weight (used to calculate body mass index—BMI) [28];
3. Personal status (single, married, divorced, widowed);
4. Number of children and age of the youngest child;
5. Receiving pharmaceutical treatment (yes/no), if yes—please specify;
6. Working (yes/no) and type of work (daytime, night-time, shifts);
7. Waking up during the night (yes/no), if yes—how many times per night.

#### 2.1.2. Symptoms of Nocturnal Masticatory Muscle Activity

Questions referring to symptoms of nocturnal masticatory activity, as follows:

1. Possible sleep bruxism (SB) was evaluated through the question: “Are you aware, or have you been told, that you grind or clench your teeth when you are asleep?” (yes/no) [29]. This methodology is valid for screening for bruxism among large populations [30,31,32]; it adheres to the definition of “possible” bruxism, as graded by the consensus papers on bruxism [27,33];

2. Experiencing headache upon awakening (headache; yes/no);

3. Experiencing stiffness of the oral and/or neck musculature upon awakening (muscle stiffness; yes/no). The question was derived from the TMD-pain screener questionnaire [34], which was confirmed by the International Network for Orofacial Pain and Related Disorders Methodology (INfORM), as adhering to the INfORM standards [35].

#### 2.1.3. Risk of OSA

The Arabic version of the STOP-BANG Questionnaire, developed and validated by Chung et al. [36] as a screening tool for OSA, was used in the present study. The questionnaire showed high-sensitivity, especially for patients with moderate to severe OSA. The validity and reliability of the Arabic version of the questionnaire was published previously [37].

The STOP-BANG questionnaire is comprised of four self-report yes/no questions referring to: (i) snoring loudly (Snoring); (ii) feeling tired, fatigued, or sleepy during the daytime (Tired); (iii) have been observed by another to have stopped breathing during sleep (Observed); and (iv) being treated for high blood pressure (Pressure). The total score of the STOP-BANG questionnaire ranges 0–4.

The authors suggested an additional scoring model which combines BMI, age, neck circumference, and gender. In the present study, data regarding BMI, age, and gender (all female) were collected, as described above. The neck perimeter of all participants was measured by one of the female Arab researchers (JS or a research assistant). The measurement was carried out with a tape measure at the base of the neck, with head upright and shoulders at rest. The procedure was practiced and standardized by the two researchers on six subjects, who were not part of the final study.

Three risk categories for OSA were defined [38] as follows:

1. Low risk: score of 0–2 on the self-report questions;
2. Intermediate risk: score of 3–4 on the self-report questions;
3. High risk: score of 2–4 on the self-report questions, in addition to BMI > 35 kg/m^2^, or score of 2–4, in addition to neck perimeter > 41 cm.

In the present study, information from the STOP-BANG questionnaire was analyzed, as follows:

1. Risk categories for OSA, as specified above (OSA-risk);
2. Score of the STOP questionnaire (OSA-STOP);
3. The question regarding snoring (yes/no), as a separate variable (Snoring).

#### 2.1.4. Daytime Sleepiness (Sleepiness)

Daytime sleepiness was assessed through the Epworth sleepiness scale [39]. This is a simple, self-administered questionnaire, which provides a measurement of the subject’s general level of daytime sleepiness. The scale is widely used to evaluate daytime sleepiness, particularly among patients with SDB.

The questionnaire consists of eight items, scored on a scale between 1 and 4. It evaluates daytime sleepiness through questions regarding the subjects’ likelihood of dozing off or falling asleep in common daily life daytime situations (e.g., reading, watching TV, talking, etc.).

The final scores of daytime sleepiness (ESS) are as follows:

1. 0–5, low normal;
2. 6–10, high normal;
3. 11–12, mildly excessive;
4. 13–15, moderately excessive;
5. 16–24, severely excessive.

### 2.2. Statistical Analysis

Data were analyzed using SPSS software (IBM SPSS statistics 27.0, Armonk, NY, USA). In the first step, chi-square and *t*-test analyses were used to compare between the groups, with regard to personal data (age, BMI, neck circumference, personal status, number of children, age of youngest child, type of work), day sleepiness, OSA-STOP, SB, headache, and muscle stiffness upon awakening. 

In the second step, Pearson correlation coefficients, chi-square, and ANOVA analyses were used to look for possible relationships among the study variables.

## 3. Results

### 3.1. Study Population

A total of 254 women completed the study questionnaires, as follows:

Group 1 (EarlyA)—134 women completed the study questionnaires and had their neck perimeter measured.

As some of the women in the EarlyA group had newborn babies, a fact which might have affected their night sleep, women whose youngest child was at the age of ≤1 years (a typical 1-year-old sleeps 10–12 h at night without waking [40]), and women who were pregnant, were excluded from the study (n = 22). The final number of participants in the EarlyA group was 112.

Group 2 (MiddleA)—120 women in this age group completed the study questionnaires and had their neck perimeter measured. Women who were receiving hormone therapy (N = 4) were excluded from the study. The final number of participants in the MiddleA group was 116.

### 3.2. Comparisons between the EarlyA and the MiddleA Groups

As expected, the groups differed in age and in the age-related variables of BMI, number of children, and age of youngest child (Table 1). The groups differed with regard to subjects’ matrimonial status (32.1% unmarried in the EarlyA group vs. 2.6% in the MiddleA group, *p* < 0.000, chi-square). 

The older MiddleA group was taking more medications, mostly against excessive blood pressure and/or elevated cholesterol levels (31.9% in the MiddleA vs. 3.6% in the younger EarlyA group, *p* < 0.000, chi-square). In the EarlyA group, 92.9% of women worked outside home vs. only 58.8% of the MiddleA women (*p* < 0.000, chi-square), with no differences in their work pattern (daytime/night-time/shifts).

Significant differences between groups were observed in the neck perimeter and in the OSA-STOP score. No differences were detected in daytime sleepiness scores between the groups (Table 1).

The older MiddleA group reported more waking up periods during the night (61.2% of the MiddleA group reported waking up “sometimes” compared to 33.0% among the EarlyA group, *p* < 0.000, chi-square). 

There were no differences between groups, as far as headaches and possible SB are concerned, but the groups differed with regard to muscle stiffness upon awakening (as many as 63.4% of the EarlyA group experienced no muscle stiffness vs. only 50.0% among the MiddleA group, *p* < 0.05, chi-square).

The risk of OSA (OSA-risk) was significantly higher among the MiddleA group, with 5.2% of the MiddleA group being at high risk of OSA, compared to only 1.8% of their younger, EarlyA, counterparts (*p* < 0.05, chi-square, Table 2).

There were no differences between groups in their risk for daytime sleepiness.

### 3.3. Comparisons between Snoring and Non-Snoring Subjects

Out of the 228 women who were included in the analyses, 55 (24%) reported snoring. There were significant differences between the snoring and not snoring subjects regarding age, BMI, number of children, age of youngest child, neck circumference, and OSA-STOP, but not regarding daytime sleepiness (Table 3).

A significantly higher proportion of women who snore reported waking up at night, compared to the non-snoring women (85% vs. 69%, chi-square, *p* < 0.01). Snoring women experienced more headaches (33.3% vs. 19.3%, chi-square, *p* < 0.05), more muscle stiffness upon awakening (34.3% vs. 16.3%, chi-square, *p* < 0.005), and more SB (35.8% vs. 20.6%, chi-square, *p* < 0.05), compared to non-snoring women. In total, 11% of the snoring women showed high risk for OSA, compared to only 1% among the non-snoring group (chi-square, *p* < 0.000).

## 4. Discussion

SB is associated with various comorbidities, including OSA, insomnia, and others [41,42,43,44]. The SB periods of teeth grinding are characterized by specific behaviors of the mandible and stereotypical mandibular jaw movements [45,46]. Clinically, subjects with SB experience cervical pain upon awakening and morning headaches more frequently than non-bruxing individuals [47].

Although diagnosis of a definite SB is complicated (e.g., use of polysomnography), self-reported questionnaires are a common tool for screening for SB among various populations [29,30,31,32]. Symptoms of nocturnal masticatory activity, such as muscle stiffness and headache upon awakening, can serve as additional indicators of possible SB.

The OSA syndrome may carry numerous adverse consequences. It is associated with an increased likelihood of hypertension, cardiovascular disease, stroke, daytime sleepiness, motor-vehicle accidents, and diminished quality of life [48]. OSA is more common in men than in women, while in women it is more common among post-menopausal women, compared to their younger, pre-menopausal counterparts [48]. OSA is often characterized by loud, frequent snoring and daytime sleepiness. Some of the physical symptoms involved include obesity, large neck circumference, and oro-dental findings [49].

The present study was performed on two groups of women who differed significantly in age and age-related variables, such as matrimonial status, number of children, age of youngest child, working outside home, BMI, neck circumference, and use of medications. Despite the vast demographic differences between the groups, the younger women did not differ from their older counterparts in terms of SB or headaches. The only symptom of nocturnal masticatory muscle activity, which showed a difference between the two age groups, was muscle stiffness upon awakening. Nevertheless, the older women showed a significantly higher risk of OSA than the younger group.

In line with previous reports, the findings confirm that prevalence of OSA in women varies throughout life span [50]. The disparity of OSA prevalence in women, compared to men, is not only due to different pathophysiologic factors, but also to the under-recognition of sleep-disordered breathing in women due to atypical presentation and difference in polysomnographic phenotypes [50].

The main symptoms of OSA are snoring and excessive daytime sleepiness [50]. In the present study, women who reported snoring were older, had higher BMI, larger neck circumference, higher scores on the STOP-BANG questionnaire, and showed higher risk of OSA. However, they did not show higher daytime sleepiness. Franklin et al. [51] showed that sleep apnea in females is not related to daytime sleepiness. Baldwin et al. [52] suggested that men and women answer questions on sleepiness differently and that the ESS questionnaire seems to be a more sensitive measure of subjective sleepiness in men than in women. Apparently, the ESS might not be a suitable indicator of OSA in women.

The older middle-aged group reported waking up more frequently during the night, more headaches, muscle stiffness upon awakening, and more possible SB. Ohayon et al. [53] showed that subjects with OSA (odds ratio 1.8) and loud snorers (odds ratio 1.4) are at higher risk of reporting SB. Thus, signs of nocturnal masticatory muscle activity, such as headache, muscle stiffness upon awakening, and sleep bruxism should raise concerns regarding a possibility of OSA. 

Snoring is often regarded as a “masculine” phenomenon [54]. Women tend to under-report snoring and daytime sleepiness [3]. Their report is often less direct and refers to non-specific symptoms of SDB, such as headache, fatigue, depression, anxiety, sleep onset insomnia, and sleep disruption [11,12]. The present results confirm that reports of nocturnal masticatory muscle activity among middle-aged (most probably post-menopausal) women should not be discarded lightly, but, instead, considered as a possible sign of a more serious underlying condition such as OSA.

Practice guidelines published by the American Academy of Sleep Medicine and the American Academy of Dental Sleep Medicine [55,56,57] claim that OSA should not be diagnosed by dentists [56,58]. Nevertheless, Lobezzo et al. [59] showed that dental sleep disorders, such as bruxism, sleep-related orofacial pain, xerostomia, and other, regularly occur as comorbidities and are rarely found in isolation in any one individual patient. Therefore, even if the final diagnosis of OSA cannot be reached in the dental office, raising patients’ awareness to the possibility of OSA and referring them for further examination, is of importance.

Dentists are among the most often visited caregivers in the community who focus their examination on the orofacial area. Expanding the focus of dental diagnosis beyond the oral cavity and paying attention to common daily symptoms, such as SB, snoring, muscle stiffness, or headaches upon awakening, can help dentists to detect initial signs of OSA. This is especially important in middle-aged women who might not be aware of the medical risks involved and who often do not volunteer such information to their caregivers. It is the dentists’ responsibility to pay attention to oral signs of possible SB and include questions about snoring, morning muscle stiffness, and headaches upon awakening in their intake. When a concern of OSA arises, the STOP-BANG questionnaire should be used. Such simple procedures can help dentists to identify subjects at high risk of OSA and refer them to a sleep specialist.

## 5. Conclusions

Taking into account the health risks associated with untreated OSA, dentists should develop higher awareness to possible signs of SB and OSA, especially among their middle-aged female patients.

Caregivers in general and dentists in particular, should actively inquire about SB, snoring, and signs of nocturnal masticatory activity among their patients. If their findings raise concerns for the presence of OSA risk, the patient should be referred for further evaluation. 

## 6. Limitations

The study was performed on two groups of Arab women who are not necessarily representative of their (or other) age groups. The study groups were defined only by chronological age, with no information about subjects’ physiological age and/or hormonal status. Evaluations were carried out through self-report with no clinical examinations, which could confirm myalgia and/or the presence of probable SB. Oral conditions, such as malocclusions and/or temporomandibular disorders, which may affect the evaluated variables, were not considered. 

Further studies, performed on larger groups and more diverse populations, could expand our understanding of the signs and symptoms which indicate the development of OSA in both females and males. Future clinical implications should include development of designated mode(s) of OSA evaluation for dental patients, which will become part of standard examination procedures. Once validated, accepted, and clinically used, such tool(s) would have an important beneficial effect on public health.

## Figures and Tables

**Table 1:** Comparisons between the EarlyA and MiddleA groups (*t*-test).

	Age Groups	EarlyA *	MiddleA	*p*
Parameters				
**Age**		33.10 ± 5.33	60.27 ± 4.31	**0.000**
BMI **		24.30 ± 4.67	27.82 ± 4.02	**0.000**
**No. of Children**		2.67 ± 0.97	4.45 ± 1.92	**0.000**
**Age of youngest child**		5.59 ± 3.47	24.43 ± 6.09	**0.000**
**Neck (cm)**		33.74 ± 3.53	36.65 ± 3.53	**0.000**
OSA-STOP ***		0.47 ± 0.75	1.18 ± 1.10	**0.000**
**Daytime Sleepiness (Total score)**		8.33 ± 4.39	7.62 ± 4.68	N.S.

* Mean ± SD, ** BMI—Body Mass index [28], *** OSA-STOP—score of the STOP questionnaire.

**Table 2:** OSA-risk among the EarlyA and MiddleA groups.

	Age Groups	EarlyA **	MiddleA
OSA Risk *			
**Low risk**		94.4%	85.3%
**Intermediate Risk**		1.8%	**9.5%**
**High Risk**		1.8%	**5.2%**

* OSA risk—risk categories of OSA, ** % within Group.

**Table 3:** Comparisons between snoring and not snoring participants (*t*-test).

	Snoring Groups	Non-Snoring *	Snoring	*p*
Parameters				
**Age**		44.13 ± 14.09	55.69 ± 11.86	**0.000**
**BMI** **		25.39 ± 4.59	28.56 ± 4.12	**0.000**
**No. of Children**		3.47 ± 1.54	4.51 ± 2.28	**0.001**
**Age of youngest child**		15.24 ± 10.32	22.53 ± 9.30	**0.000**
**Neck (cm)**		34.58 ± 3.33	37.25 ± 4.28	**0.000**
**OSA-STOP** ***		0.41 ± 0.60	2.14 ± 0.91	**0.000**
**Daytime Sleepiness (Total score)**		7.77 ± 4.27	8.60 ± 5.31	N.S.

* Mean ± SD, ** BMI—Body Mass index [28], *** OSA-STOP—score of the STOP questionnaire.

## References

[1] Deary V., Ellis J.G., Wilson J.A., Coulter C., Barclay N.L. (2014). Simple snoring: Not quite so simple after all?. Sleep Med. Rev..

[2] Young T., Palta M., Dempsey J., Skatrud J., Weber S., Badr S. (1993). The occurrence of sleep-disordered breathing among middle-aged adults. N. Engl. J. Med..

[3] Redline S., Kamp K., Tishler P.V., Browner I., Ferrette V. (1994). Gender differences in sleep disordered breathing in a community-based sample. Am. J. Respir. Crit. Care Med..

[4] Tietjens J.R., Claman D., Kezirian E.J., De Marco T., Mirzayan A., Sadroonri B., Goldberg A.N., Long C., Gerstenfeld E.P., Yeghiazarians Y. (2019). Obstructive Sleep Apnea in Cardiovascular Disease: A Review of the Literature and Proposed Multidisciplinary Clinical Management Strategy. J. Am. Heart Assoc..

[5] Appleton S., Gill T., Taylor A., McEvoy D., Shi Z., Hill C., Reynolds A., Adams R. (2018). Influence of Gender on Associations of Obstructive Sleep Apnea Symptoms with Chronic Conditions and Quality of Life. Int. J. Environ. Res. Public Health.

[6] Young T., Peppard P.E., Gottlieb D.J. (2002). Epidemiology of obstructive sleep apnea: A population health perspective. Am. J. Respir. Crit. Care Med..

[7] Bixler E.O., Vgontzas A.N., Lin H.M., Ten Have T., Rein J., Vela-Bueno A., Kales A. (2001). Prevalence of sleep-disordered breathing in women: Effects of gender. Am. J. Respir. Crit. Care Med..

[8] Dancey D.R., Hanly P.J., Soong C., Lee B., Hoffstein V. (2001). Impact of menopause on the prevalence and severity of sleep apnea. Chest.

[9] Young T., Finn L., Austin D., Peterson A. (2003). Menopausal status and sleep disordered breathing in the Wisconsin Sleep Cohort Study. Am. J. Respir. Crit. Care Med..

[10] Hall M.H., Matthews K.A., Kravitz H.M., Gold E.B., Buysse D.J., Bromberger J.T., Owens J.F., Sowers M. (2009). Race and financial strain are independent correlates of sleep-in midlife women: The SWAN sleep study. Sleep.

[11] Durán J., Esnaola S., Rubio R., Iztueta A. (2001). Obstructive sleep apnea-hypopnea and related clinical features in a population-based sample of subjects aged 30 to 70 yr. Am. J. Respir. Crit. Care Med..

[12] Shepertycky M.R., Banno K., Kryger M.H. (2005). Differences between men and women in the clinical presentation of patients diagnosed with obstructive sleep apnea syndrome. Sleep.

[13] Badran M., Yassin B.A., Fox N., Laher I., Ayas N. (2015). Epidemiology of Sleep Disturbances and Cardiovascular Consequences. Can. J. Cardiol..

[14] Hermann D.M., Bassetti C.L. (2016). Role of sleep-disordered breathing and sleep-wake disturbances for stroke and stroke recovery. Neurology.

[15] Schillaci G., Battista F., Fiorenzano G., Basili M.C., Crapa M., Alrashdi Y., Pucci G. (2015). Obstructive Sleep Apnea and Cardiovascular Disease—A New Target for Treatment. Curr. Pharm. Des..

[16] Lu M., Wang Z., Zhan X., Wei Y. (2021). Obstructive sleep apnea increases the risk of cardiovascular damage: A systematic review and meta-analysis of imaging studies. Syst. Rev..

[17] Ayas N.T., Taylor C.M., Laher I. (2016). Cardiovascular consequences of obstructive sleep apnea. Curr. Opin. Cardiol..

[18] Karunanayake C., Dosman J., Fenton M., Rennie D., Kirychuk S., Ramsden V., Seeseequasis J., Abonyi S., Pahwa P. (2019). Association between Co-Morbidities and the Prevalence of Excessive Daytime Sleepiness over a Four-Year Period. Clocks Sleep.

[19] Omobomi O., Batool-Anwar S., Quan S.F. (2019). Clinical and Polysomnographic Correlates of Subjective Sleepiness in Mild Obstructive Sleep Apnea. Sleep Vigil..

[20] Balasubramaniam R., Klasser G.D., Cistulli P.A., Lavigne G.J. (2014). The Link between Sleep Bruxism, Sleep Disordered Breathing and Temporomandibular Disorders: An Evidence-Based Review. JDSM.

[21] Lavigne G.J., Khoury S., Abe S., Yamaguchi T., Raphael K. (2008). Bruxism physiology and pathology: An overview for clinicians. J. Oral Rehabil..

[22] Solanki N., Singh B.P., Chand P., Siddharth R., Arya D., Kumar L. (2017). Effect of mandibular advancement device on sleep bruxism score and sleep quality. J. Prosthet. Dent..

[23] Phillips B.A., Okeson J., Paesani D., Gilmore R. (1986). Effect of sleep position on sleep apnea and parafunctional activity. Chest.

[24] Kato T., Katase T., Yamashita S., Sugita H., Muraki H., Mikami A., Okura M., Ohi M., Masuda Y., Taniguchi M. (2013). Responsiveness of jaw motor activation to arousals during sleep in patients with obstructive sleep apnea syndrome. J. Clin. Sleep Med..

[25] Khoury S., Rouleau G.A., Rompre P.H., Mayer P., Montplaisir J.Y., Lavigne G.J. (2008). A significant increase in breathing amplitude precedes sleep bruxism. Chest.

[26] Manfredini D., Guarda-Nardini L., Marchese-Ragona R., Lobbezoo F. (2015). Theories on possible temporal relationships between sleep bruxism and obstructive sleep apnea events: An expert opinion. Sleep Breath..

[27] Lobbezoo F., Ahlberg J., Raphael K.G., Wetselaar P., Glaros A.G., Kato T., Santiago V., Winocur E., De Laat A., De Leeuw R. (2018). International consensus on the assessment of bruxism: Report of a work in progress. J. Oral Rehabil..

[28] https://www.thecalculatorsite.com/articles/health/bmi-formula-for-bmi-calculations.php.

[29] https://www.iadr.org/Portals/69/docs/Groups/INfORM/Oral-Behavior-Checklist_2013-05-12.pdf.

[30] Winocur E., Messer T., Eli I., Emodi-Perlman A., Kedem R., Reiter S., Friedman-Rubin P. (2019). Awake and sleep bruxism among Israeli adolescents. Front. Neurol..

[31] Emodi Perlman A., Lobbezoo F., Zar A., Friedman-Rubin P., van Selms M.K., Winocur E. (2016). Self-reported bruxism and associated factors in Israeli adolescents. J. Oral Rehabil..

[32] van Selms M.K., Visscher C.M., Naeije M., Lobbezoo F. (2013). Bruxism and associated factors among Dutch adolescents. Community Dent. Oral Epidemiol..

[33] Lobbezoo F., Ahlberg J., Glaros A.G., Kato T., Koyano K., Lavigne G.J., De Leeuw R., Manfredini D., Svensson P., Winocur E. (2013). Bruxism defined and graded: An international consensus. J. Oral Rehabil..

[34] Gonzalez Y.M., Schiffman E., Gordon S.M., Seago B., Truelove E.L., Slade G., Ohrbach R. (2011). Development of a brief and effective temporomandibular disorder pain screening questionnaire: Reliability and validity. J. Am. Dent. Assoc..

[35] International RDC-TMD Consortium|A Consortium Fostering the Evidence-Based Diagnosis & Management of Orofacial Pain and Jaw Disorders (Buffalo.Edu). https://ubwp.buffalo.edu/rdc-tmdinternational/wp-content/uploads/sites/58/2017/01/TMD-Pain-Screener_revised-10Aug2011.pdf.

[36] Chung F., Yegneswaran B., Liao P., Chung S.A., Vairavanathan S., Islam S., Khajehdehi A., Shapiro C.M. (2008). STOP Questionnaire: A Tool to Screen Patients for Obstructive Sleep Apnea. Anesthesiology.

[37] Bahammam A.S., Al-Aqeel A., Alhedyani A., Al-Obaid G.H., Al-Owais M., Olaish A. (2015). The validity and reliability of an Arabic version of the STOP-Bang questionnaire for identifying obstructive sleep apnea. Open Respir. Med. J..

[38] The Official Site of Stop-Bang Questionnaire. http://www.stopbang.ca/osa/results.php.

[39] Johns M.W. (1991). A new method for measuring daytime sleepiness: The Epworth sleepiness scale. Sleep.

[40] Bathory E., Tomopoulos S. (2017). Sleep Regulation, Physiology and Development, Sleep Duration and Patterns, and Sleep Hygiene in Infants, Toddlers, and Preschool-Age Children. Curr. Probl. Pediatr. Adolesc. Health Care.

[41] da CostaLopes A.J., Cunha T.C.A., Monteiro M.C.M., Serra-Negra J.M., Cabral L.C., Júnior P.C.S. (2020). Is there an association between sleep bruxism and obstructive sleep apnea syndrome? A systematic review. Sleep Breath..

[42] Tan M.W.Y., Yap A.U., Chua A.P., Wong J.C.M., Parot M.V.J., Tan K.B.C. (2019). Prevalence of sleep bruxism and its association with obstructive sleep apnea in adult patients: A retrospective polysomnographic investigation. J. Oral Facial Pain Headache.

[43] Maluly M., Dal Fabbro C., Andersen M.L., Herrero Babiloni A., Lavigne G.J., Tufik S. (2020). Sleep bruxism and its associations with insomnia and OSA in the general population of Sao Paulo. Sleep Med..

[44] Kuang B., Li D., Lobbezoo F., de Vries R., Hilgevoord A., de Vries N., Huynh N., Lavigne G., Aarab G. (2022). Associations between sleep bruxism and other sleep-related disorders in adults: A systematic review. Sleep Med..

[45] Martinot J.B., Borel J.C., Le-Dong N.N., Silkoff P.E., Denison S., Gozal D., Pépin J.L. (2020). Bruxism relieved under CPAP treatment in a patient with OSA syndrome. Chest.

[46] Martinot J.B., Le-Dong N.N., Cuthbert V., Denison S., Gozal D., Lavigne G., Pépin J.L. (2021). Artificial Intelligence Analysis of Mandibular Movements Enables Accurate Detection of Phasic Sleep Bruxism in OSA Patients: A Pilot Study. Nat. Sci. Sleep.

[47] Molina O.F., Simião B., Junior F.F., Peixoto M.S., Ogawa W.N., Soares F. (2018). Cervical Pain on Awakeningin the Morning Andbruxing Behavior Types: A Comparison Study and Prelimi-nary Results. IOSR J. Dent. Med. Sci..

[48] Lin C.M., Davidson T.M., Ancoli-Israel S. (2008). Gender differences in obstructive sleep apnea and treatment implications. Sleep Med. Rev..

[49] Kato T., Yamaguchi T., Okura K., Abe S., Lavigne G.J. (2013). Sleep less and bite more: Sleep disorders associated with occlusal loads during sleep. J. Prosthodont. Res..

[50] Ayub S., Won C.H.J. (2019). Obstructive Sleep Apnea in Women. J. Sleep Med..

[51] Franklin K.A., Sahlin C., Stenlund H., Lindberg E. (2013). Sleep apnoea is a common occurrence in females. Eur. Respir. J..

[52] Baldwin C.M., Kapur V.K., Holberg C.J., Rosen C., Nieto F.J., Sleep Heart Health Study Group (2004). Associations between gender and measures of daytime somnolence in the sleep heart health study. Sleep.

[53] Ohayon M.M., Li K.K., Guilleminault C. (2001). Risk factors for sleep bruxism in the general population. Chest.

[54] Westreich R., Gozlan-Talmor A., Geva-Robinson S., Schlaeffer-Yosef T., Slutsky T., Chen-Hendel E., Braiman D., Sherf Y., Arotsker N., Abu-Fraiha Y. (2019). The Presence of Snoring as Well as its Intensity Is Underreported by Women. J. Clin. Sleep Med..

[55] American Academy of Sleep Medicine, American Academy of Dental Sleep Medicine Policy Statement on the Diagnosis and Treatment of OSA. American Academy of Dental Sleep Medicine Website. Published 7 December 2012. https://aadsm.org/docs/jointpolicy.pdf.

[56] Ramar K., Dort L.C., Katz S.G., Lettieri C.J., Harrod C.G., Thomas S.M., Chervin R.D. (2015). Clinical practice guideline for the treatment of obstructive sleep apnea and snoring with oral appliance therapy: An update for 2015. J. Clin. Sleep Med..

[57] American Academy of Dental Sleep Medicine AADSM Treatment Protocol: Oral Appliance Therapy for Sleep Disordered Breathing: An Update for 2013. American Academy of Dental Sleep Medicine Website. Published June 2013. https://aadsm.org/docs/Treatment_Protocol_FINAL.pdf.

[58] American Academy of Dental Sleep Medicine Position Paper on Portable Monitoring. American Academy of Dental Sleep Medicine Website. Published August 2005. https://pubmed.ncbi.nlm.nih.gov/16307299/.

[59] Lobbezoo F., de Vries N., de Lange J., Aarab G. (2020). A Further Introduction to Dental Sleep Medicine. Nat. Sci. Sleep.

